# Mitogen-Activated Protein Kinases 3/6 Reduce Auxin Signaling via Stabilizing Indoleacetic Acid-Induced Proteins 8/9 in Plant Abiotic Stress Adaptation

**DOI:** 10.3390/ijms26051964

**Published:** 2025-02-24

**Authors:** Chunyan Wang, Xiaoxuan Li, Han Zhao, Xiankui Cui, Wenhong Xu, Ke Li, Yang Xu, Zipeng Yu, Luyao Yu, Rui Guo

**Affiliations:** 1 The Key Laboratory of Plant Development and Environmental Adaptation Biology, Ministry of Education, School of Life Sciences, Shandong University, Qingdao 266237, China; wangchunyan5127@163.com (C.W.); lixiaoxuan0105@163.com (X.L.); 13280869871@163.com (H.Z.); qdcuixk@126.com (X.C.); xuwenhong_123@163.com (W.X.); yuzipeng@sdu.edu.cn (Z.Y.); 2 Shandong Academy of Grape, Shandong Academy of Agricultural Sciences, Jinan 250100, China; like_sdu@foxmail.com; 3 Shandong Peanut Research Institute, Shandong Academy of Agricultural Sciences, Qingdao 266100, China; xy52120092661@163.com

**Keywords:** auxin signaling, stress adaptation, plant growth balance, MPK3/6, IAA8/9, phosphorylation, protein stability

## Abstract

The balance between plant growth and stress response is a key issue in the field of biology. In this process, mitogen-activated protein kinase 3 (MPK3) and MPK6 contribute to the construction of plants’ defense system during stress tolerance, while auxin, a growth-promoting hormone, is the key to maintaining plant growth. Nevertheless, the antagonistic or cooperative relationship between MPK3/6-mediated stress response and auxin-mediated plant growth remains unclear. Here, we demonstrate that stress-activated MPK3/6 interact with the auxin signaling repressors indoleacetic acid-induced protein 8 (IAA8) and IAA9, two key targets for regulating the auxin signaling output during stress responses. Protein phosphorylation mass spectrometry followed by a co-analysis with in vitro phosphorylation experiments revealed that MPK3/6 phosphorylated the S91, T94, and S152 residues of IAA8 and the S88 residue of IAA9. Phosphorylation significantly enhanced the protein stability of IAA8/9, thereby maintaining basal auxin signaling during plant stress adaptation. Collectively, MPK3/6-IAA8/9 are key modules that are turned on during plant stress adaptation to precisely reduce auxin signaling output, thereby preventing plants from improper vigorous growth under stress conditions.

## 1. Introduction

Nearly half of crop yield losses are attributed to environmental pressures [1], including abiotic stresses (such as drought, extreme temperatures, and high salinity) and biotic stresses (such as pathogen and insect infections). For example, high salinity affects leaf and shoot growth, seed germination, crop productivity, disrupted metabolism, and growth inhibition [1,2]. Drought stress induces poor growth performance in plants, decreases leaf water content, reduces cellular turgor pressure, and lowers the transpiration rate [3]. To survive and reproduce in constantly changing environments, plants must precisely balance their growth and stress responses [4]. When confronted with abiotic stresses, plants slow down their growth and evolve specific adaptations to allow growth by reprogramming their gene-regulatory networks [5,6,7]. The mechanism by which plants coordinate the balance between external environmental signals and their intrinsic growth signals to respond to abiotic stresses remains the object of in-depth investigations.

As a key signaling module downstream of receptor-like kinases (RLKs), the mitogen-activated protein (MAP) kinase cascades function as molecular switches in perceiving various upstream stress or developmental signals, ultimately regulating plant growth or stress adaptation [8]. A typical MAPK cascade signaling module consists of MAP kinase kinase kinase (MAP3K, MKKK, or MEKK), MAP kinase kinase (MAP2K, MKK, or MEK), and MAP kinase (MAPK or MPK) [9]. There are 80 MKKKs, 10 MKKs, and 20 MPKs in Arabidopsis based on similarities in the amino acid sequence [10]. MPK3 and MPK6 are instrumental in plant immunity and stress responses [11]. Moreover, MPK3/6 play pivotal roles in almost all aspects of plant growth and development, including gametophyte development, embryogenesis, senescence, hypocotyl elongation, lateral root formation, and organ abscission [8,12,13]. Therefore, MPK3/6 is a potential balancing factor for plant growth and stress response.

As a growth-promoting phytohormone, auxin plays an important role in plant growth and development, including root growth [14,15], hypocotyl elongation [13], organogenesis [16], lateral root formation [17,18], and stress responses [7,10]. The core components of the canonical auxin signaling pathway comprise auxin receptors such as transport inhibitor-resistant 1 (TIR1) and auxin signaling F-BOXs (AFBs), the transcriptional repressors called auxin/indole-3-3 acetic acids (Aux/IAAs), and the transcription factors called auxin response factors (ARFs) [19]. Under low-auxin conditions, Aux/IAAs interact with ARFs and inhibit their transcriptional activity, whereas auxin facilitates the binding of Aux/IAAs into TIR1/AFBs, leading to the ubiquitination and degradation of Aux/IAAs, thereby removing inhibition on ARFs-regulated auxin-responsive genes [19]. Importantly, MPKs are also vital players in auxin-regulated plant growth, such as root stem cell niche identity, hypocotyl elongation, and lateral root formation [12,13,15,17,20].

Nevertheless, how MPK3/6, as a stress-adapting factor, and auxin, as a growth-promoting factor, cooperate or antagonize to enhance stress adaptation in plants remains a mystery. In this study, we found the interactions between MPK3/6 and the auxin signaling repressors IAA8 and IAA9. We also demonstrated that stress-activated MPK3/6 phosphorylate IAA8 at the S91, T94, and S152 residues and phosphorylate IAA9 at the S88 residue, thereby enhancing protein stabilization. In summary, this study suggests that MPK3/6 downregulate auxin signaling in plants by stabilizing the IAA8/9 proteins in plant abiotic stress adaptation.

## 2. Results

### 2.1. Auxin Signaling Output Is Tightly Controlled in Plant Abiotic Stress Responses

**Figure 1:**
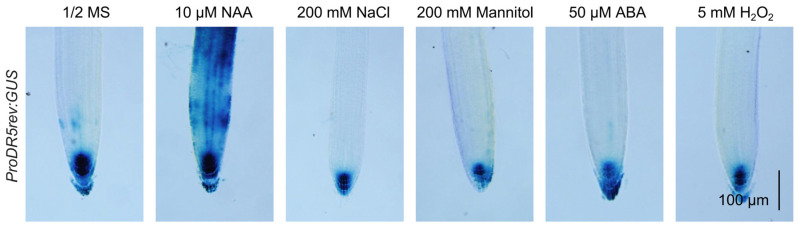
Abiotic stresses repress auxin signaling. Histochemical β-glucuronidase (GUS) staining with 7-day-old transgenic plants harboring the auxin-responsive reporter gene *DR5:GUS* treated with 200 mM NaCl, 200 mM mannitol, 50 μM ABA, 5 mM H_2_O_2_, or 10 μM NAA. Scale bar, 100 µm. NAA, 1-naphthaleneacetic acid.

Auxin is well established as the primary regulator of plant growth and development [19], positioning it as a likely intermediary in stress-reduced plant growth. As expected, using the *DR5:GUS* reporter system as an auxin signaling response marker, we observed that 1-naphthaleneacetic acid (NAA) significantly enhanced histochemical GUS staining signals in roots, while other stress simulations, including NaCl, abscisic acid (ABA), H_2_O_2_, and mannitol, reduced GUS signals in roots (Figure 1). These results are consistent with the previous speculation that the growth-promoting hormone auxin may be a key node hormone that is tightly regulated in plant stress adaptation [7]. Reduced auxin signaling output leads to basal growth of plants under stress conditions, which may be a key strategy for plant tolerance to stress.

### 2.2. MPK3/6 Interact with Many Auxin Signaling Components

**Figure 2:**
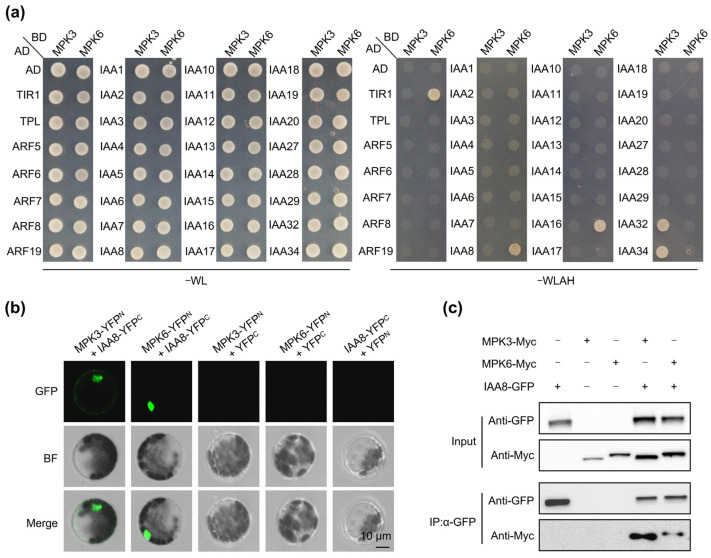
MPK3/6 interact with IAA8. (**a**) Yeast two-hybrid (Y2H) assay, showing the interactions between MPK3/6 and TIR1, Aux/IAAs, TPL, and ARFs. (**b**) Bimolecular fluorescence complementation (BiFC) assay performed in Arabidopsis protoplasts, detecting the interactions between MPK3/6 and IAA8. Scale bar, 10 μm. (**c**) Co-immunoprecipitation (Co-IP) assays performed in Arabidopsis protoplasts, validating the interactions between MPK3/6 and IAA8. IP was performed with an anti-GFP antibody, and interactions with MPK3/6-Myc were detected with an anti-Myc antibody.

As key factors in plant defense during stress adaptation, MPK3/6 are potential explanations for reduced auxin signaling output under stress conditions. Excitingly, Zhu et al. observed enhanced *DR5:GUS* signals in the double-mutants *MPK3SR* (*mpk3 mpk6 MPK3pro:MPK3^TG^*) and *MPK6SR* (*mpk3 mpk6 MPK6pro:MPK6^TG^*), as well as in the double-mutant *mkk4 mkk5*, strongly supporting our inference [21]. Therefore, we examined the interactions between MPK3/6 and various auxin signaling components, including the auxin receptor transport inhibitor response 1 (TIR1), auxin signaling repressors auxin/indole-3-3 acetic acids (Aux/IAAs), Aux/IAA-interacting protein TOPLESS (TPL), and the core auxin-activated transcription factors called auxin response factors (ARFs). Our yeast two-hybrid (Y2H) assays performed in yeasts, bimolecular fluorescence complementation (BiFC) assays in Arabidopsis protoplasts, and co-immunoprecipitation (Co-IP) experiments in Arabidopsis protoplasts revealed that MPK3/6 interact with TIR1, IAA8, IAA16, IAA32, and IAA34 (Figure 2 and Figure S1). transport inhibitor response 1.

Four of the five MPK3/6-interacting proteins belonged to the Aux/IAA family, implying that Aux/IAAs may be key targets for MPK3/6 regulation of auxin signaling. Therefore, Aux/IAAs became our main point of interest and were selected for further molecular mechanism elucidation. Nonetheless, although IAA32 and IAA34 are potential MPK3/6 phosphorylation targets, *IAA32* and *IAA34* are specifically expressed in the apical hook and regulate the formation of the apical hook [22]. In this study, we mainly sought to elucidate the molecular mechanism by which MPK3/6 reduce auxin signaling in roots under stress conditions. Therefore, we temporarily excluded IAA32/34 and focused on IAA8/16 in this study.

### 2.3. MPK3/6 Phosphorylate IAA8 at S91, T94, and S152 Residues

**Figure 3:**
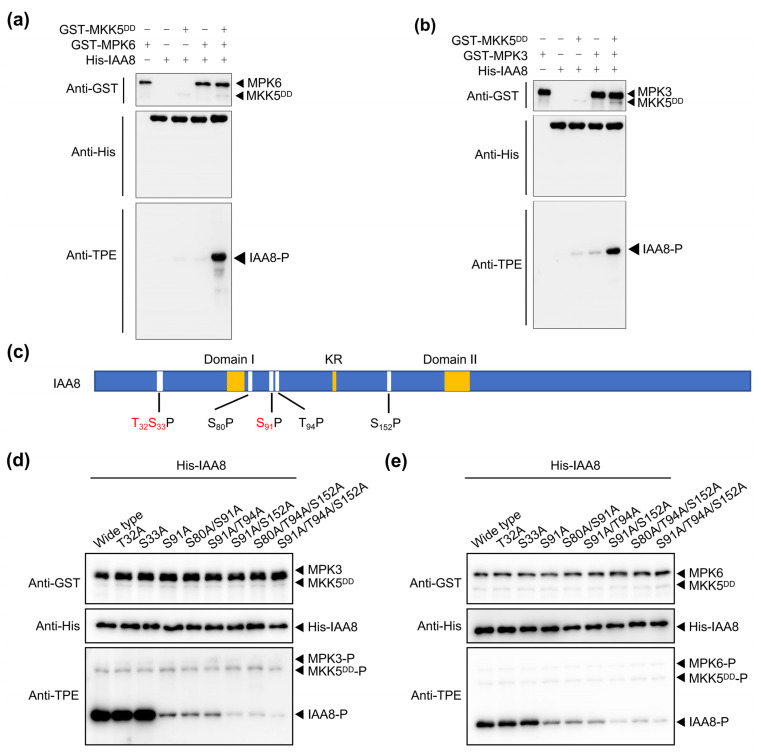
MPK3/6 phosphorylate IAA8. (**a**,**b**) In vitro kinase assays showing whether MPK3/6, which are activated by MKK5^DD^, phosphorylate IAA8. The anti-thiophosphate ester-specific (anti-TPE) antibody was used to visualize the phosphorylated proteins. The anti-GST or anti-His antibody was used to determine whether the loading quantity of each protein was consistent. (**c**) The schematic diagram illustrates the potential TP/SP motifs within the IAA8 that may be phosphorylated by MPK3/6. (**d**,**e**) The candidate phosphorylation sites in IAA8 were confirmed using an in vitro phosphorylation assay.

Since stress-activated MPK3/6 interact with IAA8/16 (Figure 2 and Figure S1), we hypothesized that MPK3/6 might phosphorylate IAA8/16, thereby reducing auxin signaling. To explore this possibility, we purified recombinant glutathione S-transferase (GST)-tagged proteins GST-MKK5^DD^, GST-MPK3, and GST-MPK6 and histidine (His)-tagged proteins His-IAA8 and His-IAA16 in *E. coli* BL21 cells and performed in vitro phosphorylation assays. The anti-thiophosphate ester-specific antibody (anti-TPE), which specifically recognizes phosphorylated proteins [13,23,24], revealed the phosphorylation of MPK6 and IAA8 in the presence of MKK5^DD^ (constitutionally activated MKK5, used to activate MPK3/6), while MPK6 alone did not phosphorylate IAA8 (Figure 3a). We obtained the same results with MPK3 (Figure 3b), indicating that MKK5-activated MPK3 and MPK6 in turn phosphorylated IAA8. Unlike the intense phosphorylation of IAA8, the MKK5^DD^-MPK3/6 module could not phosphorylate IAA16 (Figure 3a,b and Figure S2), suggesting that IAA8, not IAA16, is the node where MPK3/6 regulate auxin signaling under stress conditions.

To further identify the IAA8 site(s) phosphorylated by MPK3/6, we performed a phosphorylation mass spectrometry assay. Three potential phosphorylation sites (T32, S33, and S91) within the amino acid sequence of IAA8 were identified (Figure 3c and Figure S3). Then, we mutated T32, S33, or S91 to the non-phosphorylated form residue alanine in IAA8^T32A^, IAA8^S33A^, or IAA8^S91A^, respectively, and repeated the in vitro phosphorylation assays. Mutation of T32 or S33 did not affect the IAA8 phosphorylation level, whereas mutation of the S91 residue remarkably reduced the phosphorylation level of IAA8 (Figure 3d,e), indicating that S91 was the main phosphorylation residue phosphorylated by MPK3/6. Although the phosphorylation intensity of IAA8^S91A^ was significantly reduced, it could still be phosphorylated by MPK3/6, indicating that S91 was not the only phosphorylation site (Figure 3d,e).

Since MPK3/6 recognize typical serine–proline (SP) and threonine–proline (TP) motifs [13,25], we searched for other potential MPK3/6 recognition motifs in the IAA8 protein sequence and successfully obtained three motifs: S80P, T94P, and S152P (Figure 3c). On the basis of S91A, we performed various combinations of mutations at these three sites, and the results of in vitro phosphorylation experiments showed that T94 and S152 residues could also be phosphorylated by MPK3/6 (Figure 3d,e). In summary, MPK3/6 phosphorylated the S91, T94, and S152 residues of IAA8, among which S91 was the primary residue.

### 2.4. MPK3/6-Mediated Phosphorylation Stabilizes IAA8

**Figure 4:**
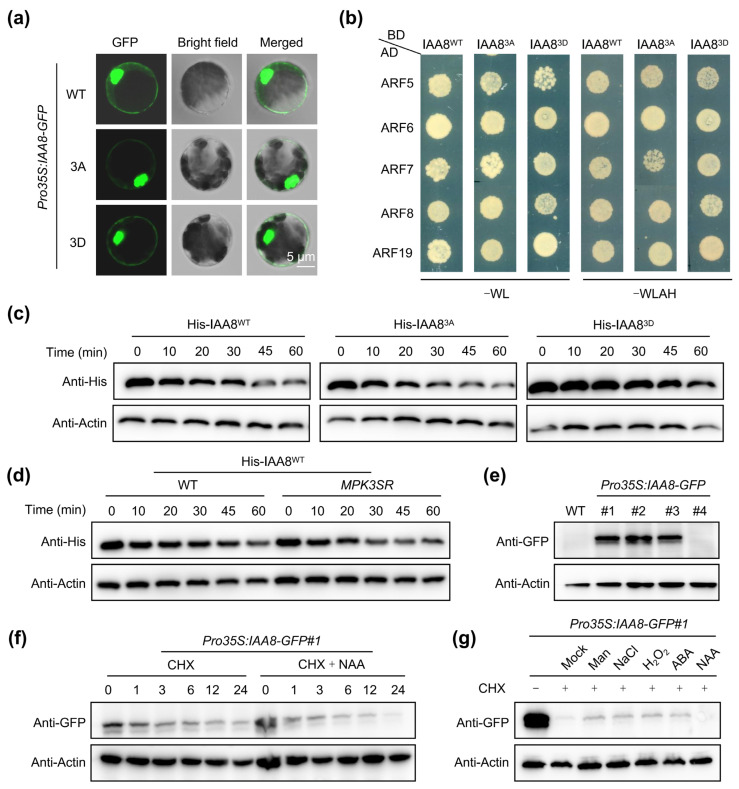
MPK3/6-mediated phosphorylation stabilizes IAA8. (**a**) Confocal images of GFP signals in Arabidopsis protoplasts expressing IAA8^WT^-GFP, IAA8^3D^-GFP, or IAA8^3A^-GFP. (**b**) Y2H assay validating the interaction between IAA8^WT^, IAA8^3A^, or IAA8^3D^ and the auxin-activated ARFs. (**c**) Degradation of the recombinant His-IAA8^WT^, His-IAA8^3A^, and His-IAA8^3D^ in the protein extracts from 8-day-old WT seedlings in cell-free assays. (**d**) Degradation of His-IAA8^WT^ in the protein extracts from 8-day-old WT seedlings or *MPK3SR* seedlings in cell-free assays. (**e**) Protein level of IAA8 in different *Pro35S:IAA8-GFP* transgenic lines. (**f**) Protein degradation of IAA8 in *Pro35S:IAA8-GFP*#1 seedlings treated with or without NAA. (**g**) Protein degradation of IAA8 in *Pro35S:IAA8-GFP*#1 seedlings treated with or without various treatments. Seedlings were treated with 125 μM CHX or 125 μM CHX + 200 mM NaCl, 200 mM mannitol, 50 μM ABA, 5 mM H_2_O_2_, and 10 μM NAA for 6 h. CHX, cycloheximide.

Phosphorylation on the target protein usually influences its cellular localization, interaction intensity with other proteins, protein/DNA-binding ability, enzymatic activity, or protein stability [10,12,17,20,26]. To detect whether the MPK3/6-mediated phosphorylation of IAA8 affects its cellular localization, we expressed wild-type IAA8 (IAA8^WT^-GFP), a phosphomimic IAA8 variant, whereby the S91, T94, and S152 residues were mutated to aspartic acid (D) in IAA8^3D^, and a non-phosphorylated IAA8 variant, whereby the S91, T94, and S152 residues were mutated to alanine (A) in IAA8^3A^ in Arabidopsis mesophyll protoplast cells, respectively. We found that the phosphorylation state of IAA8 did not affect its cellular localization (Figure 4a). Importantly, Aux/IAAs interact with ARFs and then repress the transcription of the ARF-regulated genes [19,27], promoting us to test whether MPK3/6-mediated phosphorylation of IAA8 affected its interaction with ARFs. In the Y2H experiment, IAA8^WT^, IAA8^3A^, and IAA8^3D^ showed no difference in the interaction intensity with the auxin-activated ARFs, including ARF5, ARF6, ARF7, ARF8, and ARF19 (Figure 4b), indicating that MPK3/6-mediated phosphorylation did not affect the interaction between IAA8 and ARFs.

To further investigate whether this phosphorylation modification affected the protein stability of IAA8, we incubated and calculated the degradation rates of the recombinant His-IAA8^WT^, His-IAA8^3A^, and His-IAA8^3D^ in the protein extracts from WT seedlings in cell-free assays. Compared with His-IAA8^WT^, the degradation of His-IAA8^3A^ was significantly elevated, whereas His-IAA8^3D^ was very stable (Figure 4c), suggesting that phosphorylation modification stabilized the IAA8 protein. Furthermore, we incubated the recombinant His-IAA8^WT^ in the protein extracts from WT and *MPK3SR*, whereby the embryo lethality of the *mpk3 mpk6* double-mutant was rescued by a version of MPK3 whose activity was repressed by 4-amino-1-tert-butyl-3-(1′-naphthyl) pyrazolo[3,4-d] pyrimidine (NA-PP1) [13,28]. We found that the His-IAA8^WT^ proteins were more unstable in the protein extracts from *MPK3SR* seedlings than in those from WT (Figure 4d). Collectively, MPK3/6-mediated phosphorylation enhanced the protein stability of IAA8.

As a negative regulator of auxin signaling, IAA8 was degraded in the presence of NAA (Figure 4e,f), confirming the importance of its protein quantity for auxin signaling output. Stress-activated MPK3/6 significantly enhanced the protein stability of IAA8, prompting us to further investigate the effects of various stresses on the protein stability of IAA8. Not surprisingly, multiple treatments significantly increased the protein stability of IAA8 (Figure 4g). These results suggest that abiotic stresses reduce auxin signaling in plants by stabilizing the auxin signaling repressor IAA8, a process which relies on phosphorylation modification mediated by two crucial kinases, MPK3 and MPK6.

### 2.5. MPK6 Phosphorylates IAA9 at S88 Residue

**Figure 5:**
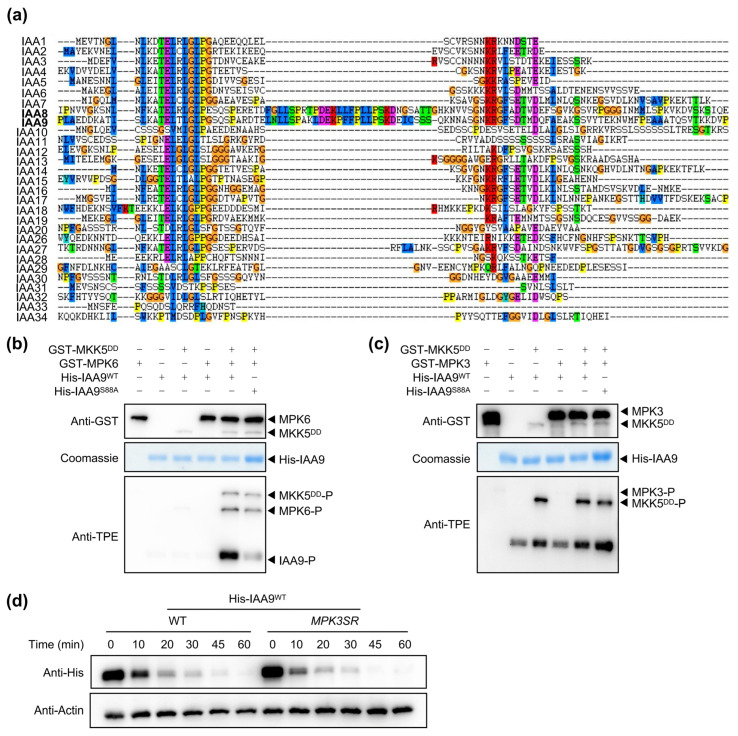
MPK6 phosphorylates and enhances the protein stability of IAA9. (**a**) Amino acid sequence alignments of IAAs in Arabidopsis. (**b**,**c**) In vitro kinase assays showing whether MPK3/6, which are activated by MKK5^DD^, phosphorylate IAA9. The anti-TPE antibody was used to visualize phosphorylated proteins. The anti-GST or anti-His antibody was used to determine whether the loading quantity of each protein was consistent. (**d**) Protein degradation of the recombinant His-IAA9^WT^ in the protein extracts from 8-day-old WT seedlings or *MPK3SR* seedlings in cell-free assay.

IAA9 shares the closest homology with IAA8, and protein sequence alignment reveals that an SP motif (S88P) in IAA9 is homologous to the SP motif (S91P, the key phosphorylation residue) in IAA8 (Figure 5a). This motif is only observed in IAA8 and IAA9 (Figure 5a), suggesting that MPK3/6 might also phosphorylate IAA9. Subsequently, we performed an in vitro phosphorylation assay and found that MPK6, but not MPK3, could phosphorylate IAA9 (Figure 5b,c). Furthermore, mutation of the S88 residue to Ala (IAA9^S88A^) significantly reduced the phosphorylation of IAA9 mediated by MPK6 (Figure 5b), indicating that S88 was the major site for the MPK6-mediated phosphorylation of IAA9.

Homologous proteins IAA8 and IAA9 share the same phosphorylation site, suggesting that MPK-mediated phosphorylation may have the same effect on IAA8 and IAA9, namely enhancing their protein stability. Then, we incubated the recombinant His-IAA9^WT^ in the protein extracts from WT and *MPK3SR* seedlings in the cell-free assays. The degradation rate of His-IAA9^WT^ was faster in *MPK3SR* than in the WT (Figure 5d), indicating that MPK6 enhanced the protein stability of IAA9.

## 3. Discussion

Abiotic stresses, such as high salinity, low temperature, and drought, seriously affect plant growth and crop production [29]. When faced with abiotic stresses, plants slow down their growth and evolve specific adaptations to allow growth by reprogramming their gene-regulatory networks [5,6]. The plant hormone auxin plays a crucial role in plant growth and development, and previous studies have shown that abiotic stresses affect auxin synthesis, transport, and signaling transduction [30,31,32]. In this study, we showed that various abiotic stresses inhibited auxin signaling (Figure 1), confirming the speculation that abiotic stresses suppress auxin signaling [7].

A previous study demonstrated that mIAA8, a functionally gain-of-function mutant of IAA8, interacts with ARF6/8 to inhibit their transcriptional activity, subsequently reducing the production of jasmonic acid (JA) and resulting in defects in floral organ development [33]. The MPK-mediated phosphorylation of IAA8 disturbs the expression of flower development-related genes to inhibit flower development under heat stress [34]. Cold signals and reactive oxygen species (ROS) promote the accumulation of IAA8 to inhibit the transcriptional activity of ARFs, and, in turn, IAA8 represses the transcription of ABA Insensitive 3 (ABI3) to facilitate seed germination [35]. Here, we revealed that various abiotic stresses reduced auxin signaling by stabilizing IAA8/9 (Figure 1 and Figure 4g), which expanded our understanding of IAA8/9 in plant response to stress. IAA8 may be the key for plants to actively reduce auxin signaling in response to stress, so the activation of MPK3/6-IAA8/9 modules may be necessary for plant stress adaptation.

MPK3/6 are essential for both plant growth and stress response [25,30,34,36,37,38,39], making them critical for balancing these two functions. Importantly, MPK3/6 can phosphorylate IAA15 to regulate lateral root formation under drought stress conditions [39] and can also phosphorylate IAA8 to regulate flower development under heat stress conditions [35]. Here, we found that MPK3/6 not only phosphorylate IAA8 and IAA9 but also interact with TIR1, IAA16, IAA32, and IAA34 in Y2H assays (Figure 2a), implying that there may be more substrates phosphorylated by MPK3/6. TIR1 is a component of the ubiquitin ligase (E3) complex SKP1-CUL-FBP (SCF) and receives the auxin signal to recruit Aux/IAA repressors to the SCF^TIR1^ complex for ubiquitination and degradation [7]. TIR1/AFBs also play crucial roles in response to abiotic and biotic stresses and are regulated post-translationally by other proteins [40]. Heat shock protein 90 (HSP90) interacts with and stabilizes TIR1 during plant development regulation [41]. The FERONIA receptor kinase–NADPH oxidase signaling pathway regulates the auxin-mediated oxidation of TIR1/AFB2 and their subsequent nuclear import [42]. Here, we found interactions between MPK3/6 and TIR1 (Figure 2a and Figure S1), implying that the stress-activated MPK3/6 may phosphorylate TIR1, thereby modulating the subcellular localization of TIR1. IAA32 and IAA34 belong to non-canonical Aux/IAAs, which lack a TIR1-binding domain [22]. Auxin-activated TMK1 C-terminus (kinase domain) phosphorylates IAA32 and IAA34 to regulate apical hook formation and maintenance [22]. Therefore, we hypothesized that MPK3/6 might also phosphorylate IAA32 and IAA34 to regulate apical hook development in response to abiotic stresses. Notably, MPK3/6 phosphorylate IAA9 at the S88 residue (Figure 5b), which is conserved in IAA8 at the S91 residue (Figure 5a). This residue is absent in other IAAs (Figure 5a), indicating that MPK3/6 may phosphorylate IAA32 and IAA34 at other sites. The function of MPK3/6-IAA32/34 modules in plant abiotic stress adaptation is worthy of further investigation.

**Figure 6:**
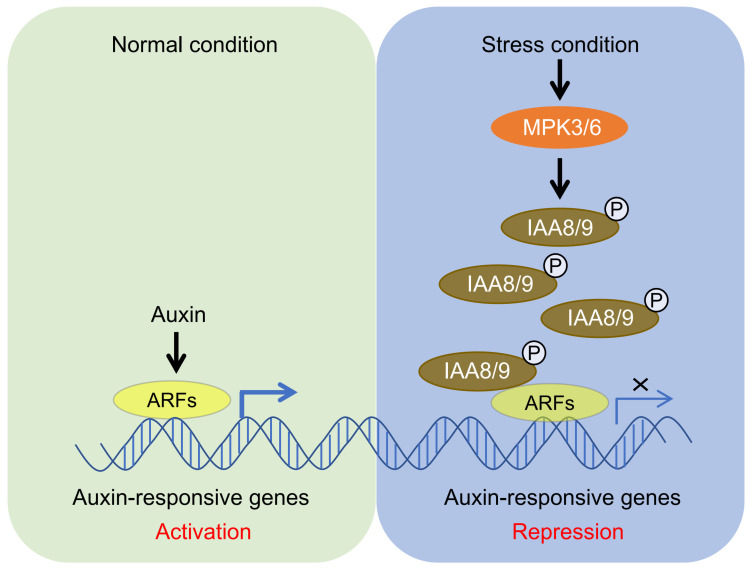
Proposed model of MPK3/6-IAA8/9 module in plant stress adaptation. Under normal conditions, as a growth-promoting hormone, auxin maintains the expression of many growth-related genes by activating the key transcription factor ARFs, thus ensuring the normal growth and development of plants. Under stress conditions, MPK3/6-mediated phosphorylation significantly enhances the protein stability of IAA8/9, resulting in basal auxin signaling for plant stress adaptation. ARF, auxin response factor; MPK3, mitogen-activated protein kinase 3; and IAA8, auxin/indole-3-3 acetic acid 8.

In summary, this study reveals that, when plants are exposed to various stresses, activated MPK3/6 phosphorylate IAA8/9 and enhance their protein stability to reduce auxin signaling in plants (Figure 6). This study provides some new perspectives to understand how plants balance plant growth and stress response by regulating MPK3/6-mediated IAA8/9 protein levels.

## 4. Materials and Methods

### 4.1. Plant Materials and Growth Conditions

All plant materials used in this study were from the *Arabidopsis thaliana* (L.) Columbia-0 background. Arabidopsis seeds from WT and transgenic lines *DR5:GUS* [14], *MPK3SR* (*mpk3 mpk6 ProMPK3:MPK3^TG^*) [28], and *Pro35S:IAA8-GFP* were used for gene expression and function analyses. *Pro35S:IAA8-GFP* was generated in this study, and the coding sequence of *IAA8* was cloned into the pSuper1300-GFP vector (Yaji Biological, Shanghai, China) to construct *Pro35S:IAA8-GFP* using the primers listed in Table S1. The floral-dip method with *Agrobacterium tumefaciens* strain GV3101 (Tsingke, Beijing, China) was used to perform the transformation of Arabidopsis, and homozygous transgenic plants were selected on half-strength MS medium (1.5% [*w*/*v*] sucrose and 0.85% [*w*/*v*] agar) supplemented with 40 mg L^−1^ hygromycin and confirmed by Western blot assays. For stress treatments, 7-day-old *DR5:GUS* transgenic lines were treated with 10 µM 1-naphthaleneacetic acid (NAA), 200 mM NaCl, 200 mM mannitol, 50 μM abscisic acid (ABA), or 5 mM H_2_O_2_ for 6 h. All selected Arabidopsis plants were grown in a controlled growth chamber under long-day conditions (16 h light/8 h dark) at 22 °C.

### 4.2. Yeast Two-Hybrid Assay (Y2H)

Protein interaction assays in yeast were performed according to the protocol for the Matchmaker Gold Yeast Two-Hybrid System (GAL4 based; Takara, Beijing, China). The coding sequences of the target genes were cloned into pGBKT7 and pGADT7 vectors and transformed into the yeast strain AH109 according to the protocol. Positive transformants were selected on a synthetic defined (SD) medium lacking leucine (Leu) and tryptophan (Trp) and grown at 30 °C for 2 days. Physical interactions were indicated by the ability of cells to grow on SD medium without Leu, Trp, His, and Ade for 2 to 3 days after plating. The primers used in the Y2H assays are listed in Table S1.

### 4.3. Bimolecular Fluorescence Complementation (BiFC) Assay in Arabidopsis Mesophyll Protoplasts

BiFC assays were performed as previously described [13]. The CDS of *MPK3* or *MPK6* was cloned into the pSAT6-nEYFP-C1 vector to construct MPK3-YFP^N^ or MPK6-YFP^N^, and the coding sequence of *TIR1* or *IAAs* was cloned into the pSAT6-cEYFP-C1 vector (Yaji Biological, Shanghai, China) to construct TIR1-YFP^C^ or IAAs-YFP^C^. The protoplasts were prepared as previously described [13]. The plasmid combinations were transfected into Arabidopsis mesophyll protoplasts and then cultured at 25 °C for 12 h in the dark. The GFP signals were detected using a confocal microscope (Zeiss LSM 900, Oberkochen, Germany) with a 488 nm laser excitation wavelength and a 500–550 nm emission wavelength. The primers used for the BiFC assay are listed in Table S1.

### 4.4. Co-Immunoprecipitation (Co-IP) Assay in Arabidopsis Mesophyll Protoplasts

A Co-IP assay was performed as previously described [17] to detect the interactions between MPKs and TIR1 or IAAs. The CDS of *MPK3*, *MPK6*, *TIR1*, or *IAA16* was cloned into the pSuper1300-Myc vector to generate MPK3-Myc, MPK6-Myc, TIR1-Myc, or IAA16-Myc; the CDS of *MPK3*, *MPK6*, or *IAA8* was cloned into the pSuper1300-GFP vector to construct MPK3-GFP, MPK6-GFP, or IAA8-GFP. The protoplasts were prepared as previously described [13]. The various plasmid combinations were co-transfected into Arabidopsis mesophyll protoplasts and then cultured at 25 °C for 12 h in the dark. The anti-GFP antibody (Chromo Tek, 1:100 dilution, Munich, Germany) was used to immunoprecipitate IAA8-GFP, and the anti-Myc antibody (ABclonal, 1:5000 dilution, Boston, MA, USA) was used to detect the protein level of MPK3-Myc or MPK6-Myc. The anti-Myc antibody (Chromo Tek, 1:100 dilution, Germany) was used to immunoprecipitate IAA16-Myc or TIR1-Myc, and the anti-GFP antibody (ABclonal, 1:5000 dilution, Boston, MA, USA) was used to detect the protein level of MPK3-GFP or MPK6-GFP. The primers used for the Co-IP assay are listed in Table S1.

### 4.5. In Vitro Kinase Assay

The in vitro kinase assay was performed as previously described [13]. The coding sequences of *GST-MKK5^T215D^*^/*S221D*^ (*MKK5^DD^*) [38], *GST-MPK6*, and *GST-MPK3* were cloned into the pGEX-4T-1 vector to add a GST tag. The CDS of *IAA8*, *IAA8^T32A^*, *IAA8^S33A^*, *IAA8^S91A^*, *IAA8^S80A^*^/*S91A*^, *IAA8^S91A^*^/*T94A*^, *IAA8^S91A^*^/*S152A*^, *IAA8^S80A^*^/*T94A*/*S152A*^, *IAA8^S91A^*^/*T94A*/*S152A*^, *IAA9*, *IAA9^S88A^*, or IAA16 was cloned into the pET30a vector to add an His tag. The production of recombinant proteins was induced in *E. coli* BL21 cells with 0.4 mM isopropyl β-D-1-thiogalactopyranoside (IPTG) at 16 °C for 12 h and purified with glutathione beads (Smart-Lifesciences, Changzhou, China) or Ni IDA beads (Smart-Lifesciences, Changzhou, China). The in vitro kinase assay was performed as described previously [36]. In brief, 0.1 μg of recombinant GST-MKK5^DD^, 0.2 μg of GST-MPK3 or GST-MPK6, and 2 μg of His-IAA8, the mutant His-IAA8, or His-IAA16 were incubated in a 30 μL reaction buffer (10 mM MgCl_2_, 1 mM adenosine 5′-O-[3-thiotriphosphate], 50 mM tris-HCl [pH 7.5], and 1 mM DTT) at 30 °C for 1 h. Then, 20 mM EDTA and 1.5 mM p-nitrobenzylmesylate (ABclonal, Boston, MA, USA) were added and incubated at 25 °C for another 1 h, and boiled samples with 5× loading buffer were separated by SDS-PAGE and analyzed by immunoblotting. The anti-TPE antibody (ABclonal, Boston, MA, USA), which specifically recognizes phosphorylated proteins [23,24], was used to detect whether MPK3 and MPK6 phosphorylated IAA8, the mutant IAA8, IAA9, the mutant IAA9, or IAA16. The anti-GST or anti-His antibody (TransGen, 1:5000 dilution, Beijing, China) was used to determine whether the loading quantity of each protein was consistent. The primers used for the in vitro kinase assay are listed in Table S1.

### 4.6. Cell-Free Assay

The cell-free assay was performed as previously described [26]. Briefly, total proteins from 7-day-old Arabidopsis WT or *MPK3SR* seedlings were extracted with the lysis buffer (25 mM Tris-HCl [pH 7.5], 10 mM NaCl, 10 mM MgCl_2_, 4 mM PMSF, 5 mM DTT, and 10 mM ATP). After centrifugations at 14,000× *g* at 4 °C, the supernatant was collected. To detect the protein degradation of His-IAA8^WT^ or His-IAA9^WT^, 100 ng recombinant protein was incubated with 100 μL of total protein at 22 °C. All mock controls used an equal amount of solvent for each drug. Boiled samples were separated by SDS-PAGE and analyzed by immunoblotting. The anti-His antibody was used to detect His-IAA8 or His-IAA9. The anti-actin antibody (ABclonal, 1:5000 dilution, Boston, MA, USA) was used to determine whether the loading quantity of each protein was consistent.

### 4.7. Protein Stability Detection

We performed a protein stability assay as previously described [26]. In brief, 7-day-old Arabidopsis *Pro35S:IAA8-GFP*#1 seedlings grown on 1/2 MS medium at 22 °C were treated with 125 μM CHX or 125 μM CHX + 10 μM NAA for 1, 3, 6, 12, and 24 h. Boiled samples were separated by SDS-PAGE and analyzed by immunoblotting. The anti-GFP antibody (TransGen Biotech, Beijing, China) was used to detect IAA8-GFP. The anti-actin antibody was used to determine whether the loading quantity of each protein was consistent.

### 4.8. β-Glucuronidase (GUS) Staining

Histochemical GUS staining was performed as previously described [14]. Briefly, the root tips of 7-day-old seedlings were incubated in a solution (100 mM sodium phosphate buffer [pH 7.2], 50 μM potassium ferricyanide, 5 mM potassium ferrocyanide, 0.1% (*v*/*v*) Triton X-100, 2 mM 5-bromo-4-chloro-3-indolyl-β-D-glucuronide [X-gluc], and 10 mM EDTA) at 37 °C for 3 h. The samples were cleared in chloral hydrate and observed with an Olympus BX53 microscope (Tokyo, Japan).

## Data Availability

The plant materials supporting the findings of this study are available from the corresponding author upon request.

## Supplementary Materials

The following supporting information can be downloaded at https://www.mdpi.com/article/10.3390/ijms26051964/s1.

## Abbreviations

The following abbreviations are used in this manuscript:

MPK3	Mitogen-activated protein kinase 3
IAA8	Indoleacetic acid-induced protein 8
Aux/IAA	Auxin/indole-3-3 acetic acid
ARF	Auxin response factor
NAA	1-naphthaleneacetic acid
ABA	Abscisic acid
JA	Jasmonic acid
RLKs	Receptor-like kinases
MAP3K	MAP kinase kinase kinase
MAP2K	MAP kinase kinase
TIR1	Transport inhibitor response 1
AFBs	Auxin signaling F-boxs
*MPK3SR*	*mpk3 mpk6 MPK3pro:MPK3^TG^*
*MPK6SR*	*mpk3 mpk6 MPK6pro:MPK6^TG^*
TPL	TOPLESS
Y2H	Yeast two-hybrid
BiFC	Bimolecular fluorescence complementation
Co-IP	Co-immunoprecipitation
GST	Glutathione S-transferase
NA-PP1	4-amino-1-tert-butyl-3-(1′-naphthyl) pyrazolo[3,4-d] pyrimidine
anti-TPE	Anti-thiophosphate ester-specific antibody
ROS	Reactive oxygen species
ABI3	ABA-insensitive 3
CHX	Cycloheximide
