# Supplementary material for: Mitogen-Activated Protein Kinases 3/6 Reduce Auxin Signaling via Stabilizing Indoleacetic Acid-Induced Proteins 8/9 in Plant Abiotic Stress Adaptation
Source: Int J Mol Sci. 2025 Feb 24;26(5):1964. doi: 10.3390/ijms26051964 (PMC11900227; doi:10.3390/ijms26051964)
Supplement: Supplementary file 1 [file ijms-26-01964-s001.zip › ijms-3454346-supplementary.pdf]

Fig. S1

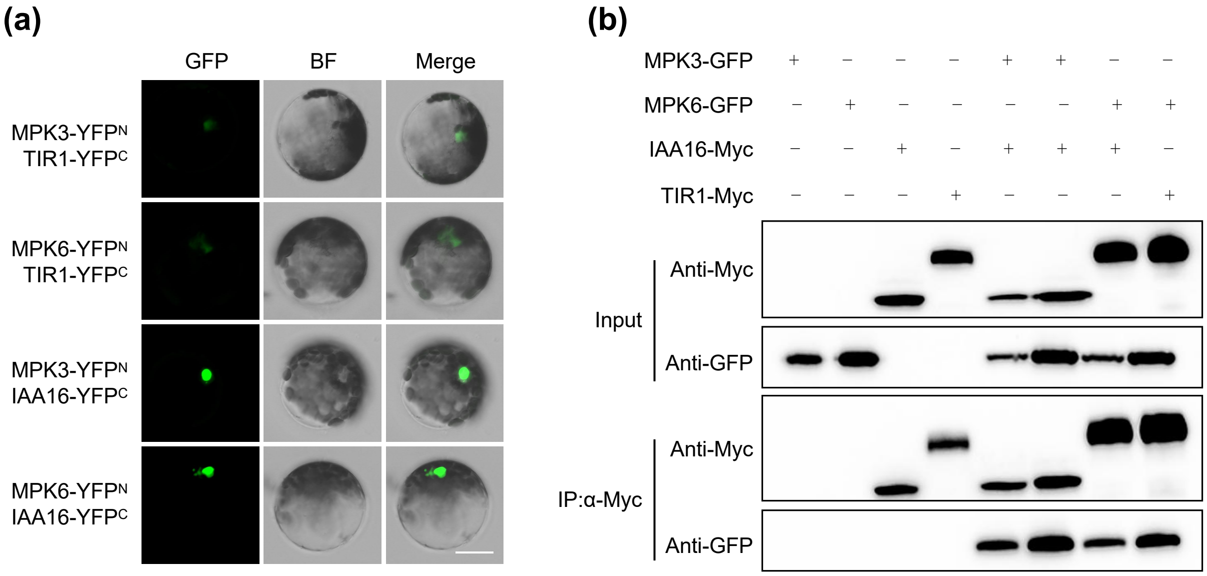

Figure S1 MPK3/6 interact with IAA16 and TIR1. **(a)** BiFC assay performed in Arabidopsis protoplasts validating the interaction between MPK3/6 and IAA16 or TIR1. Scale bar, 10 μm. **(b)** Co-IP experiments in Arabidopsis protoplasts verifying the interactions between MPK3/6 and IAA16 or TIR1. IP was performed with anti-Myc antibody, and interactions with MPK3/6-GFP were detected with anti-GFP antibody.

Fig. S2

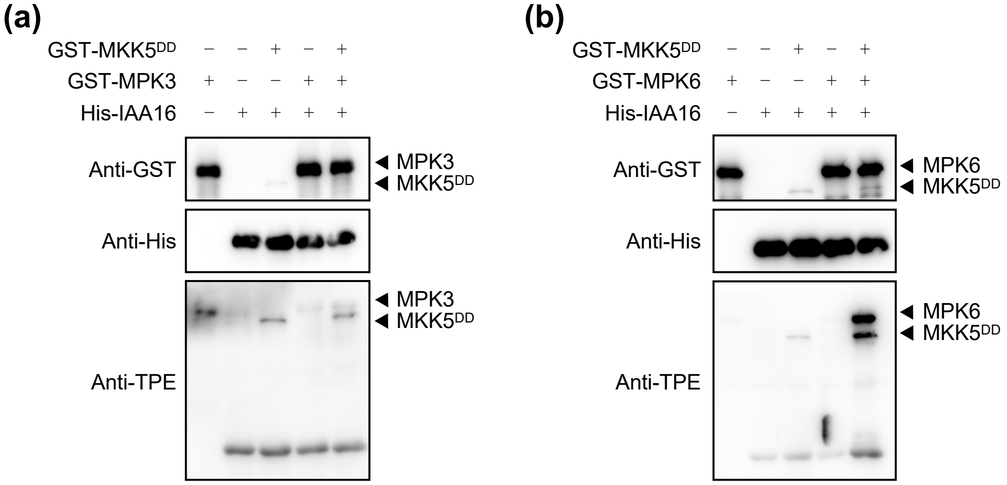

Figure S2 MPK3/6 cannot phosphorylate IAA16. (**a** and **b**) *In vitro* kinase assays showing MPK3/MPK6 which activated by $MKK5^{DD}$phosphorylate IAA16. The recombinant GST-$MKK5^{DD}$, GST-MPK3 or GST-MPK6, and His-IAA16 were incubated in reaction buffer for 1 hour. The anti–thiophosphate ester–specific antibody (anti-TPE) antibody was used to visualize phosphorylated proteins. The anti-GST or anti-His antibody was used to determine whether the loading quantity of each protein was consistent.

Fig.S3

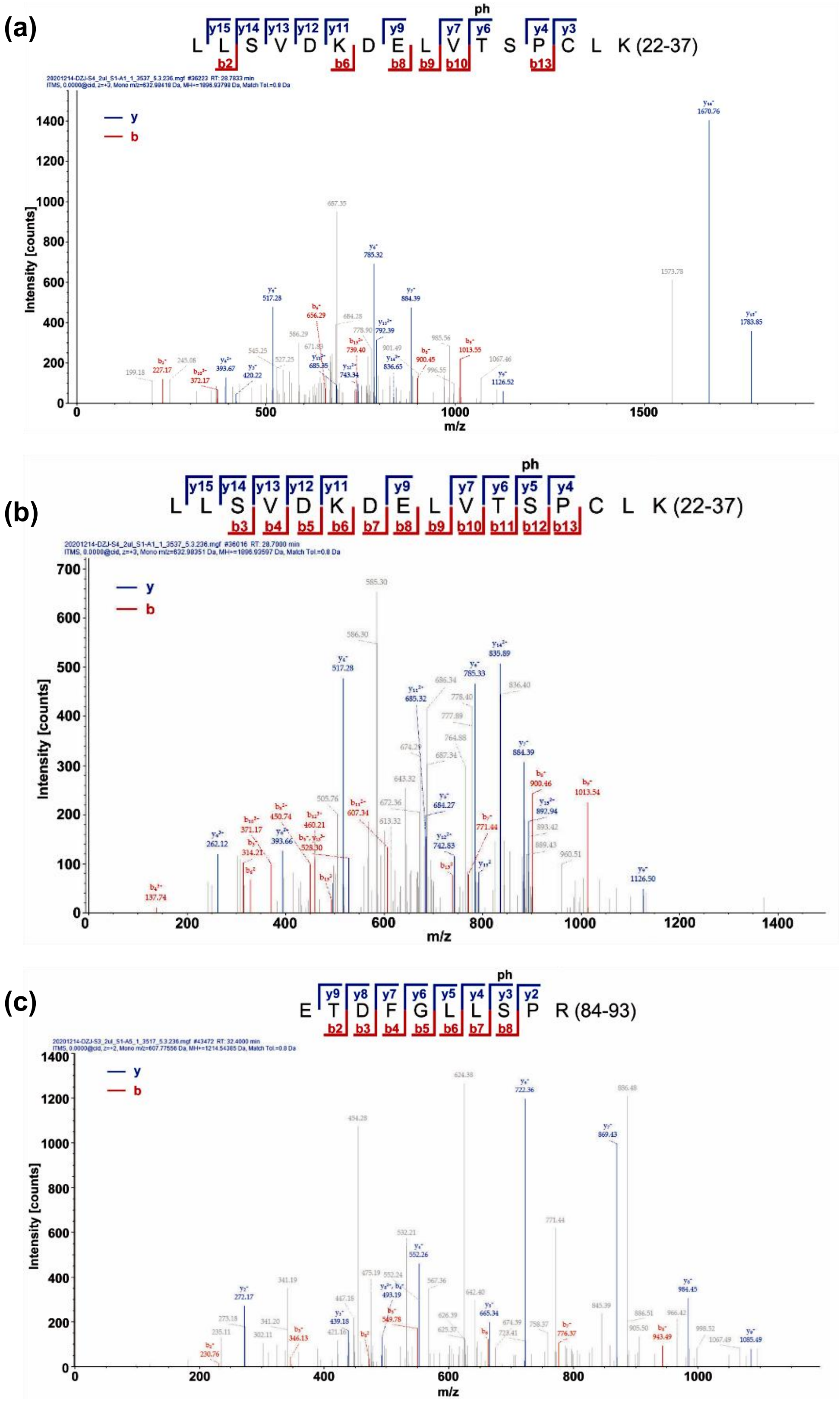

Figure S3 Identification of phosphorylation sites in IAA8. (**a-c**) The mass spectrometry peak profile displays the potential phosphorylation sites of the IAA8 phosphorylated by MPK6.

Table S1. List of primers used in this study

Prime Name	Primer sequence (from 5' to 3')
MPK3-BD-F	ATGGCCATGGAGGCCATGAACACCGGCGGTGGC
MPK3-BD-R	CCGCTGCAGGTCGACCTAACCGTATGTTGGATTGAGTGC
MPK6-BD-F	ATGGCCATGGAGGCCATGGACGGTGGTTCAGGTCA
MPK6-BD-R	CCGCTGCAGGTCGACCTATTGCTGATATTCTGGAT
TIR1-AD-F	GAGGCCAGTGAATTCATGCAGAAGCGAATAGCCTTGT
TIR1-AD-R	AGCTCGATGGATCCCTTATAATCCGTTAGTAGTAATGA
TPL-AD-F	GAGGCCAGTGAATTCATGTCTTCTCTTAGTAGAGAGCT
TPL-AD-R	AGCTCGATGGATCCCTCATCTCTGAGGCTGATCA
ARF5-AD-F	GAGGCCAGTGAATTCATGATGGCTTCATTGTCTTGTG
ARF5-AD-R	AGCTCGATGGATCCCTTATGAAACAGAAGTCTTAAG
ARF6-AD-F	GAGGCCAGTGAATTCATGAGATTATCTTCAGCTGGGT
ARF6-AD-R	AGCTCGATGGATCCCCTAGTAGTTGAATGAACCCC
ARF7-AD-F	GAGGCCAGTGAATTCATGAAAGCTCCTTCATCAAATG
ARF7-AD-R	AGCTCGATGGATCCCTCACCGGTTAAACGAAGTGGC
ARF8-AD-F	GAGGCCAGTGAATTCATGAAGCTGTCAACATCTGGA
ARF8-AD-R	AGCTCGATGGATCCCCTAGAGATGGGTCGGGTTTTGC
ARF19-AD-F	GAGGCCAGTGAATTCATGAAAGCTCCATCAAATGGAT
ARF19-AD-R	AGCTCGATGGATCCCCTATCTGTTGAAAGAAGCTGCA
IAA1-AD-F	GAGGCCAGTGAATTCATGGAAGTCACCAATGGGCTTA
IAA1-AD-R	AGCTCGATGGATCCCTCATAAGGCAGTAGGAGCTTCGGAT
IAA2-AD-F	GAGGCCAGTGAATTCATGGGCGGCCTCAAGCTTCGTGTC
IAA2-AD-R	AGCTCGATGGATCCCTCATAAGGAAGAGTCTAGAGCAGGAG
IAA3-AD-F	GAGGCCAGTGAATTCATGGGAGAAAAAGAAAGTGTC
IAA3-AD-R	AGCTCGATGGATCCCTCATACACCACAGCCTAAAC
IAA4-AD-F	GAGGCCAGTGAATTCATGGAAAAAGTTGATGTTTAT
IAA4-AD-R	AGCTCGATGGATCCCTTAAAGACCACCACAACCTAAAC
IAA5-AD-F	GAGGCCAGTGAATTCATGGCGAATGAGAGTAATAATCT
IAA5-AD-R	AGCTCGATGGATCCCTCATCCTCTGTTACATGATCTC
IAA6-AD-F	GAGGCCAGTGAATTCATGGCAAAGGAAGGTCTAGCAC
IAA6-AD-R	AGCTCGATGGATCCCTTAATCTTGCTGGAGACCAAAAC
IAA7-AD-F	GAGGCCAGTGAATTCATGATCGGCCAACTTATGAACCT
IAA7-AD-R	AGCTCGATGGATCCCTCAAGATCTGTTCTTGCAGTACTTC
IAA8-AD-F	GAGGCCAGTGAATTCATGAGTTCTGGGAACGATAAGAT
IAA8-AD-R	GCTCGAGCTCGATGGATCCCTCAAACCCGCTCTTTGTTC
IAA10-AD-F	AGCTCGATGGATCCCATGAATGGTTTGCAAGAAGT
IAA10-AD-R	AGCTCGATGGATCCCCTACTTACCTACTCCAGCTC
IAA11-AD-F	GAGGCCAGTGAATTCATGGAAGGCGGTTCCGCTAGTG
IAA11-AD-R	GCTCGAGCTCGATGGATCCCTTACAAAGAGAACATATAAC
IAA12-AD-F	GAGGCCAGTGAATTCATGCGTGGTGTGTCAGAATTG
IAA12-AD-R	AGCTCGATGGATCCCCTAAACAGGGTTGTTTCTTTGTC
IAA13-AD-F	GAGGCCAGTGAATTCATGATTACTGAACTTGAGATG
IAA13-AD-R	AGCTCGATGGATCCCCTAAACCGGCTGCTTTCGCT
continued…	continued…

Prime Name	Primer sequence (from 5' to 3')
IAA14-AD-F	GAGGCCAGTGAATTCATGAACCTTAAGGAGACGGAGCT
IAA14-AD-R	AGCTCGATGGATCCCTTATTGGAAAAACAGAAAAGAGC
IAA15-AD-F	GAGGCCAGTGAATTCATGTCACCGGAGGAATACGTTA
IAA15-AD-R	AGCTCGATGGATCCCTCACTTACATATTGTTATTATC
IAA16-AD-F	GAGGCCAGTGAATTCATGATTAATTTTGAGGCCACGGA
IAA16-AD-R	AGCTCGATGGATCCCTCAACTTCTGTTCTTGCACT
IAA17-AD-F	GAGGCCAGTGAATTCATGATGGGCAGTGTCGAGCTGA
IAA17-AD-R	AGCTCGATGGATCCCTCAAGCTCTGCTCTTGCACT
IAA18-AD-F	GAGGCCAGTGAATTCATGGAGGGTTATTCAAGAAAC
IAA18-AD-R	AGCTCGATGGATCCCTCATCTTCTCATTTTCTCTTGC
IAA19-AD-F	GAGGCCAGTGAATTCATGGAGAAGGAAGGACTCGG
IAA19-AD-R	AGCTCGATGGATCCCTCACTCGTCTACTCCTCTA
IAA20-AD-F	GAGGCCAGTGAATTCATGGGAAGAGGGAGAAGTTCATC
IAA20-AD-R	AGCTCGATGGATCCCTCAGTAGTGGTAATTAGC
IAA27-AD-F	GAGGCCAGTGAATTCATGTCTGTATCTGTAGCAGC
IAA27-AD-R	AGCTCGATGGATCCCCTAGTTCCTGCTTCTGCACTTCT
IAA28-AD-F	GAGGCCAGTGAATTCATGGAAGAAGAAAAGAGATTG
IAA28-AD-R	AGCTCGATGGATCCCCTATTCCTTGCCATGTTTTC
IAA29-AD-F	GAGGCCAGTGAATTCATGGAGTTGGATCTTGGTCTA
IAA29-AD-R	AGCTCGATGGATCCC TTAAAACAAACATCTTGTATA
IAA32-AD-F	GAGGCCAGTGAATTCATGGACCCAAACACACCTGCAG
IAA32-AD-R	AGCTCGATGGATCCCTTAAAAGGGAAGAAGAGCATCGT
IAA34-AD-F	GAGGCCAGTGAATTCATGTATTGCAGCGATCCTCCCCA
IAA34-AD-R	AGCTCGATGGATCCCTTAAAAGGGAAGTACAGCATCG
MPK3-YFP^{N}-F	CGAGCTCAAGCTTCCATGAACACCGGCGGTGGC
MPK3-YFP^{N}-R	GACTCTAGATCAGGTCTAACCGTATGTTGGATTGAGTGC
MPK6-YFP^{N}-F	CGAGCTCAAGCTTCCATGGACGGTGGTTCAGGTCA
MPK6-YFP^{N}-R	GACTCTAGATCAGGTCTATTGCTGATATTCTGGATTGAAA
IAA8-YFP^{C}-F	CGAGCTCAAGCTTCCATGAGTTCTGGGAACGATAAGAT
IAA8-YFP^{C}-R	GACTCTAGACTAGGTTCAAACCCGCTCTTTGTT
TIR1-YFP^{C}-F	CGAGCTCAAGCTTCCATGCAGAAGCGAATAGCCTTG
TIR1-YFP^{C}-R	GACTCTAGACTAGGTTAATCCGTTAGTAGTAATGATTTGCC
IAA16-YFP^{C}-F	CGAGCTCAAGCTTCCATGATTAATTTTGAGGCCACGG
IAA16-YFP^{C}-R	GACTCTAGACTAGGTTCAACTTCTGTTCTTGCACTTTTC
MPK3-Myc-F	CAAATCGACTCTAGAATGAACACCGGCGGTGGCCAATA
MPK3-Myc-R	ACTAGTATTTAAATGACCGTATGTTGGATTGAGTGCTA
MPK6-Myc-F	CAAATCGACTCTAGAATGGACGGTGGTTCAGGTCAACC
MPK6-Myc-R	ACTAGTATTTAAATGTTGCTGATATTCTGGATTGAAAG
IAA8-GFP-F	CAAATCGACTCTAGAATGAGTTCTGGGAACGATAAGAT
IAA8-GFP-R	ACTAGTATTTAAATGAACCCGCTCTTTGTT
MPK3-GFP-F	CAAATCGACTCTAGAATGAACACCGGCGGTGGCCAATA
MPK3-GFP-R	ACTAGTATTTAAATGACCGTATGTTGGATTGAGTGCTA
MPK6-GFP-F	CAAATCGACTCTAGAATGGACGGTGGTTCAGGTCAACC
MPK6-GFP-R	ACTAGTATTTAAATGTTGCTGATATTCTGGATTGAAAG
continued…	continued…

**Prime Name**	**Primer sequence (from 5' to 3')**
TIR1-Myc-F	CAAATCGACTCTAGACGACTCTAGTCTAGAATGCAGAAGCGAATAGCCTT
TIR1-Myc-R	ACTAGTATTTAAATGCATGGTACCGGATCCTAATCCGTTAGTAGTAATGATTTGC
IAA16-Myc-F	CAAATCGACTCTAGAATGATTAATTTTGAGGCCACGG
IAA16-Myc-R	ACTAGTATTTAAATGACTTCTGTTCTTGCACTTTTC
GST-MKK5^{DD}-F	CCGCGTGGATCCCCGATGAAACCGATTCAATCTCC
GST-MKK5^{DD}-R	GATGCGGCCGCTCGACTAAGAGGCAGAAGGAAGAG
GST-MPK3-F	GATGCGGCCGCTCGACTAACCGTATGTTGGATTGA
GST-MPK3-R	GATGCGGCCGCTCGACTAACCGTATGTTGGATTGA
GST-MPK6-F	CCGCGTGGATCCCCGATGGACGGTGGTTCAGGT
GST-MPK6-R	GATGCGGCCGCTCGACTATTGCTGATATTCTGGATTGA
His-IAA16-F	GCCATGGCTGATATCATGATTAATTTTGAGGCCACGG
His-IAA16-R	TGCGGCCGCAAGCTTTCAACTTCTGTTCTTGCACTTTTC
His-IAA8^{WT}-F	GCCATGGCTGATATCATGAGTTCTGGGAACGATAAGAT
His-IAA8^{WT}-R	TGCGGCCGCAAGCTTTCAAACCCGCTCTTTGTT
His-IAA8^{T32A}-F	GGATGAACTGGTTGCGTCACCTTGTTTGAAAG
His-IAA8^{T32A}-R	CTTTCAAACAAGGTGACGCAACCAGTTCATCC
His-IAA8^{S33A}-F	GGATGAACTGGTTACGGCACCTTGTTTGAAAG
His-IAA8^{S33A}-R	CTTTCAAACAAGGTGCCGTAACCAGTTCATCC
His-IAA8^{S91A}-F	GATTTCGGTTTGCTGGCTCCGAGAACACCC
His-IAA8^{S91A}-R	GGGTGTTCTCGGAGCCAGCAAACCGAAATC
His-IAA8$^{S80A/S91A}$-F	CCTGAGTCTCAAGCTCCTGAGAGAGAGACT
His-IAA8$^{S80A/S91A}$-R	AGTCTCTCTCTCAGGAGCTTGAGACTCAGG
His-IAA8$^{S91A/T94A}$-F	CGGTTTGCTGGCTCCGAGAGCACCCGATGAGAAG
His-IAA8$^{S91A/T94A}$-R	CTTCTCATCGGGTGCTCTCGGAGCCAGCAAACCG
His-IAA8$^{S91A/S152A}$-F	CAACATGATGTTGGCGCCGAAAGTTAAGG
His-IAA8$^{S91A/S152A}$-R	CCTTAACTTTCGGCGCCAACATCATGTTG
His-IAA8$^{S80A/T94A/S152A}$-F	CCTTAACTTTCGGCGCCAACATCATGTTG
His-IAA8$^{S80A/T94A/S152A}$-R	CCTTAACTTTCGGCGCCAACATCATGTTG
His-IAA8$^{S91A/T94A/S152A}$-F	CAACATGATGTTGGCGCCGAAAGTTAAGG
His-IAA8$^{S91A/T94A/S152A}$-R	CCTTAACTTTCGGCGCCAACATCATGTTG
Pro35S:IAA8^{3A}-GFP-F	CAAATCGACTCTAGAATGAGTTCTGGGAACGATAAGAT
Pro35S:IAA8^{3A}-GFP-R	ACTAGTATTTAAATGAACCCGCTCTTTGTT
Pro35S:IAA8^{3D}-GFP-F	CAAATCGACTCTAGAATGAGTTCTGGGAACGATAAGAT
Pro35S:IAA8^{3D}-GFP-R	ACTAGTATTTAAATGAACCCGCTCTTTGTT
IAA8^{3A}-BD-F	ATGGCCATGGAGGCCATGAGTTCTGGGAACGATAAGAT
IAA8^{3A}-BD-R	CCGCTGCAGGTCGACCAACCCGCTCTTTGTT
IAA8^{3D}-BD-F	ATGGCCATGGAGGCCATGAGTTCTGGGAACGATAAGAT
IAA8^{3D}-BD-R	CCGCTGCAGGTCGACCAACCCGCTCTTTGTT
His-IAA9^{WT}-F	GCCATGGCTGATATCATGTCCCCGGAAGAGGAGC
His-IAA9^{WT}-R	TGCGGCCGCAAGCTTTTAAGCTCTCATCTTCG
His-IAA9^{S88A}-F	GAGCTTAACCTTTTGGATCCAGCAAAGCTAGATG
His-IAA9^{S88A}-R	CATCTAGCTTTGCTGGATCCAAAAGGTTAAGCTC
